# The mitigative effects of *Blautia producta* 1009924 on DSS-induced intestinal inflammation

**DOI:** 10.3389/fmicb.2025.1560441

**Published:** 2025-08-08

**Authors:** Yishu Chen, Yan Ma, Yang Leng, Xiaoling Li, Sishi Qin, Xiaoyan Li, Zhao Zhang, Huajun Yu

**Affiliations:** ^1^Laboratory Animal Center, Guangdong Medical University, Zhanjiang, China; ^2^Guangdong Longseek Testing Co., Ltd., Guangzhou, China

**Keywords:** *Blautia producta* 1009924, genome, intestinal inflammation, inflammatory, short-chain fatty acids (SCFAs)

## Abstract

**Introduction:**

The gut microbiota plays a crucial role in the treatment of inflammatory bowel disease (IBD). Recent studies have shown that the abundance of *Blautia producta* is associated with host inflammatory bowel disease (IBD), yet its specific mechanism of action remains to be further investigated.

**Methods:**

The strain *Blautia producta* 1009924 (*B. producta* 1009924) was isolated from fresh fecal samples, and its biological characteristics and genomic features were analyzed. In this study, a zebrafish intestinal inflammation model was established by induction with 0.5% dextran sulfate sodium (DSS) for 72 h to evaluate the effect of *B. producta* 1009924 on alleviating intestinal inflammation.

**Results:**

This strain *B. producta* 1009924 is a strict anaerobe, forming circular, off-white colonies on BHI medium. It is Gram-positive, arranged in chains, and field emission scanning electron microscopy revealed abundant surface folds and pilus structures, along with excellent acid and bile salt tolerance. Whole-genome sequencing showed a total gene length of 6.05 Mb, a GC content of 45.72%, and 5,214 coding genes with no virulence genes. KEGG database annotation indicated that its gene functions are mainly enriched in metabolic pathways, environmental information processing, and genetic information processing, with abundant gene clusters involved in lipid metabolism and short-chain fatty acid (SCFA) metabolic pathways. Compared with the DSS-induced enteritis model group, *B. producta* 1009924 inhibited reactive oxygen species (ROS) production and neutrophil accumulation in zebrafish intestines. Histopathological analysis confirmed that it alleviated DSS-induced intestinal tissue damage, such as increasing goblet cell numbers and improving intestinal villus architecture. Real-time quantitative polymerase chain reaction (RT-qPCR) analysis demonstrated that *B. producta* 1009924 suppressed the activation of the TLR4/NF-κB signaling pathway by downregulating the expression of TLR4, MyD88, and NF-κB genes, thereby reducing the expression of pro-inflammatory factors (IL-6, IL-12) and the immune factor IL-10. Additionally, metabolomic analysis revealed that *B. producta* 1009924 regulated intestinal metabolism by increasing SCFA levels, including butyric acid and isovaleric acid.

**Conclusion:**

*Blautia producta* 1009924 significantly alleviates DSS-induced intestinal inflammation in zebrafish by regulating ROS levels, inhibiting excessive immune and inflammatory responses, and improving SCFA metabolism, highlighting its potential as a candidate strain for IBD treatment.

## 1 Introduction

The mammalian gastrointestinal tract harbors a complex and diverse community of symbiotic microorganisms, comprising bacteria, viruses, archaea, as well as eukaryotes (Zoetendal et al., 2004). As a sophisticated ecosystem, the gastrointestinal microbiome plays a critical role in regulating host energy metabolism and homeostasis, with wide-ranging impacts on host physiology—including disease progression, drug metabolism, and immune system modulation (Zoetendal et al., 2004; Wang and Yang, 2013). Notably, probiotics are defined as live microorganisms that confer health benefits by modulating the indigenous microbiota to maintain a balanced state (Chen et al., 2022).

*Blautia producta* is an anaerobic microorganism widely distributed in the intestines and feces of mammals (Liu et al., 2008, 2021), primarily isolated from metabolic products and rumen fluid. *B. producta* is a strictly anaerobic, Gram-positive, and non-motile bacterium, typically spherical or oval, occurring in pairs or chains. It grows optimally at 37°C and pH 7.0 (Lorowitz and Bryant, 1984; Liu et al., 2008). Possessing both heterotrophic and autotrophic properties, this bacterium can utilize CO, H_2_/CO_2_, and carbohydrates as energy sources (Liu et al., 2021). Thus, *B. producta* has attracted increasing attention due to its roles in alleviating inflammatory diseases and mitigating metabolic syndrome (Liu et al., 2021). As a dominant genus in the intestinal microbiota, it is closely associated with host physiological functions. A recent study by (Liu et al. 2021) has demonstrated that the composition and dynamics of *B. producta* populations are influenced by factors such as host age, geography, diet, genotype, health conditions, disease states, and other physiological status. Meanwhile, (Yang et al. 2016) reported that the relevant abundance of *B. producta* is negatively correlated with biomarkers of obesity-related metabolic disorders.

Inflammatory bowel disease (IBD) is a group of chronic, non-specific intestinal inflammatory disorders with unknown etiology, mainly including ulcerative colitis and Crohn's disease. Over the past few decades, with the acceleration of global urbanization and changes in lifestyle, the incidence of IBD has been on a continuous rise in developing countries, becoming a significant public health concern (Molodecky et al., 2012). As a key interface for the interaction between the host and the external environment, the gastrointestinal tract is a complex ecosystem composed of intestinal epithelial cells, the mucus barrier, immune cells, and intestinal microbiota. The homeostasis of this ecosystem is crucial for maintaining intestinal health. Among them, intestinal dysbiosis has been identified as one of the core factors driving the occurrence and development of enteritis, which can exacerbate intestinal inflammation by affecting immune activation, metabolic imbalance, and mucosal barrier integrity (Liu et al., 2018). Within the intestinal microbiota, *B. producta*, a potential probiotic, has attracted increasing attention in recent years due to its association with intestinal health. Existing studies have shown that *B. producta* can exert intestinal protective effects through multiple mechanisms: including directly inhibiting excessive inflammatory responses, enhancing intestinal mucosal barrier function, regulating pro-inflammatory signaling pathways such as TLR4/NF-κB, and restoring the ecological balance of intestinal microbiota, thereby effectively improving dextran sulfate sodium (DSS)-induced experimental colitis (Mao et al., 2024). Clinical studies have shown that the abundance of *B. producta* is significantly reduced in the cecal mucosal microbiota of IBD patients and the mucosa-adherent microbiota of colorectal cancer patients, suggesting that it may be involved in the pathological regulation of intestinal diseases (Chen et al., 2014, 2012).

While the abundance of *B. producta* is closely associated with various diseases, the causal relationship between this bacterium and disease pathogenesis, as well as its regulatory mechanisms and safety profiles, remains to be elucidated. Traditional probiotic research relies heavily on mammalian models, which are limited by time-consuming procedures and high experimental costs. In contrast, zebrafish have been widely used for evaluating probiotic efficacy due to their high genomic homology with humans, conserved intestinal composition, and advantageous traits such as high fecundity, transparent embryos, and suitability for high-throughput screening. In this study, *B. producta* 1009924 was isolated and screened from fecal samples. Using zebrafish as the model organism, we established an intestinal inflammation model induced by dextran sulfate sodium (DSS) to evaluate the alleviating effect of *B. producta* 1009924 on intestinal inflammation and preliminarily explore its underlying regulatory mechanisms. The findings of this study are expected to provide a theoretical basis and experimental support for the application of *B. producta* in the intervention of intestinal inflammation.

## 2 Materials and methods

### 2.1 Materials and reagents

Dextran sulfate sodium salt (DSS, D808272, Shanghai McLean Biochemical Technology Co., Ltd., China); Mesalazine (HY-15027, MedChemExpress (Monmouth Junction, USA)); ChamQ Universal SYBR qPCR Master Mix (Q711-02, Vazyme, China); FastKing one-step genomic cDNA removal first Strand synthesis master mix (KR118-02, Tiangen Biotech Co., Ltd, China); RNA Easy Fast animal tissue/cell total RNA extraction kit (DP451, Tiangen Biotech Co., Ltd, China); DCFH-DA (D6883, Sigma-Aldrich Corporation, United States). All reagents of high-performance liquid chromatography (HPLC) grade and short-chain fatty acid metabolite standards were purchased from Sigma-Aldrich Corporation.

### 2.2 *B. producta* culture medium

The liquid culture medium (brain heart infusion [BHI]) was composed of 12 g/L sucrose, 22 g/L yeast extract powder, 0.5 g/L K_2_HPO_4_, 0.5 g/L KH_2_PO_4_, 4 g/L NaHCO_3_, 1.8 g/L soybean oligosaccharides, and 1.2 g/L cysteine hydrochloride. Distilled water was then added to a final volume of 1,000 mL, and the medium was autoclaved at 121°C for 20 min.

The solid culture medium was prepared by incorporating 1.5~2.0% (w/v) agar powder into the liquid culture medium.

For the BHI–HCL culture medium, 0.1 mol/L HCl solution was added to the liquid medium to adjust the pH to 2 and 3.

For the BHI–HCL culture medium, bovine bile salt solution was added to the liquid medium to achieve mass fractions of 0.1%, 0.3%, and 0.5%.

### 2.3 Isolation, purification, and identification of *B. producta* 1009924

Fresh fecal specimens were serially diluted in sterile physiological saline within an anaerobic workstation. A 100 μL aliquot of the 10^−5^ dilution was plated onto BHI agar, followed by anaerobic incubation at 37°C with 65% relative humidity and 0.0% oxygen tension for 48 h. Isolated single colonies were further streaked onto BHI agar and anaerobically incubated for 24 h. After multiple rounds of isolation and purification, clonal colonies with consistent morphological features were obtained. A single colony from the final purification step was picked using an inoculating loop and resuspended in 20 μL of sterile water to serve as the polymerase chain reaction (PCR) template. The 16S ribosomal DNA (rDNA) gene of strain 1009924 was amplified using universal primers (27F: AGAGTTTGATCCTGGCTCAG; 1492R: TACGGCTACCTTGTTACGACTT). The obtained 16S rDNA sequence was deposited in the GenBank database of the National Center for Biotechnology Information (NCBI) and subjected to homology searches and alignments using the Basic Local Alignment Search Tool (BLAST) (Xing et al., 2022).

### 2.4 Analysis of growth characteristics and acid/bile salt tolerance of *B. producta* 1009924

Bacterial suspension was inoculated onto BHI agar and anaerobically cultured for 12 h. Single colonies were picked and inoculated into BHI broth for 24 h anaerobic incubation to prepare fermentation seed cultures. For growth curve determination, seed cultures were inoculated into BHI broth at 2% (v/v) with 3 replicates, followed by 24 h anaerobic incubation. Samples were collected every 2 h to measure OD600, and growth curves were plotted. For survival assays, seed cultures were inoculated at 1% (v/v) into BHI-HCl medium and BHI bile salt medium, respectively, with an initial viable count of 1 × 108 CFU/mL. After anaerobic incubation, viable bacteria were counted at 0, 3, and 6 h (Huang et al., 2020), and survival rates were calculated using Equation 1:


(1) $$\begin{array}{l} \text{Survival rate }\left(\text{\%}\right)=\frac{N_{1}}{N_{0}}\times 100\% \end{array}$$

In Equation 1: *N*_1_ represents the viable count measured at 3 and 6 h, in *l*g CFU/mL; *N*_0_ represents the viable count of *B. producta* 1009924 at 0 h, in *lg* CFU/mL.

### 2.5 Cell morphology analysis of *B. producta* 1009924

*B. producta* 1009924 cultures in the logarithmic growth phase were centrifuged at 5,000 *g* for 10 min at 4°C. After discarding the supernatant, the bacterial pellets were resuspended in pre-cooled 0.1 mol/L phosphate-buffered saline (PBS) buffer, washed twice, and collected. The bacterial cells were fixed in 2.5% glutaraldehyde solution at room temperature for 3 h, then rinsed three times with 0.1 mol/L PBS. Subsequent fixation was performed in 1% osmium tetroxide solution at 4°C for 2 h, followed by two additional rinses with PBS. Gradient dehydration was conducted sequentially using ethanol solutions: 30% ethanol for 10 min, 50% ethanol for 10 min, 70% ethanol for 10 min, 90% ethanol for 10 min, and 100% ethanol twice (15 min per treatment). After dehydration, the samples were dried in a critical-point dryer, coated using an ion sputter coater, and then observed and imaged under a scanning electron microscope (Kokkinosa et al., 1998).

### 2.6 Genome sequencing, assembly, structure prediction, and functional annotation of *B. producta* 1009924

Cultures of *B. producta* 1009924 in the logarithmic growth phase were centrifuged at 8,000 *g* for 10 min at 4°C to harvest bacterial cells. Genomic DNA was extracted using the cetyltrimethylammonium bromide (CTAB) method combined with magnetic bead purification. Whole-genome sequencing was performed using a hybrid approach with the Illumina NovaSeq 6000 (PE 150 mode) and PacBio Sequel II platforms (McCarthy, 2010). For PacBio sequencing, genomic DNA was fragmented to 10–15 kb using a g-TUBE device, and SMRTbell CCS libraries were constructed (average read length ≥ 15 kb). For Illumina sequencing, libraries with 350 bp insert sizes were prepared (sequencing depth ≥ 100 ×). Library quality control and quantification were conducted using a Qubit 3.0 Fluorometer (Invitrogen, Carlsbad, CA, USA) and an Agilent 2100 Bioanalyzer. PacBio HiFi reads were *de novo* assembled using Hifiasm/Canu (Nurk et al., 2020), with Canu assisting in error correction, followed by iterative polishing with Pilon using Illumina short reads. Protein-coding genes were predicted using Prodigal/Augustus (Delcher et al., 2007). Functional annotation was performed by aligning predicted proteins against the NCBI NR database, assigning Gene Ontology (GO) terms (Harris et al., 2004), mapping to Kyoto Encyclopedia of Genes and Genomes (KEGG) pathways (Ogata et al., 1999), and classifying into Clusters of Orthologous Groups (COG).

### 2.7 Maintenance and treatment of zebrafish larvae

**Figure 1:**
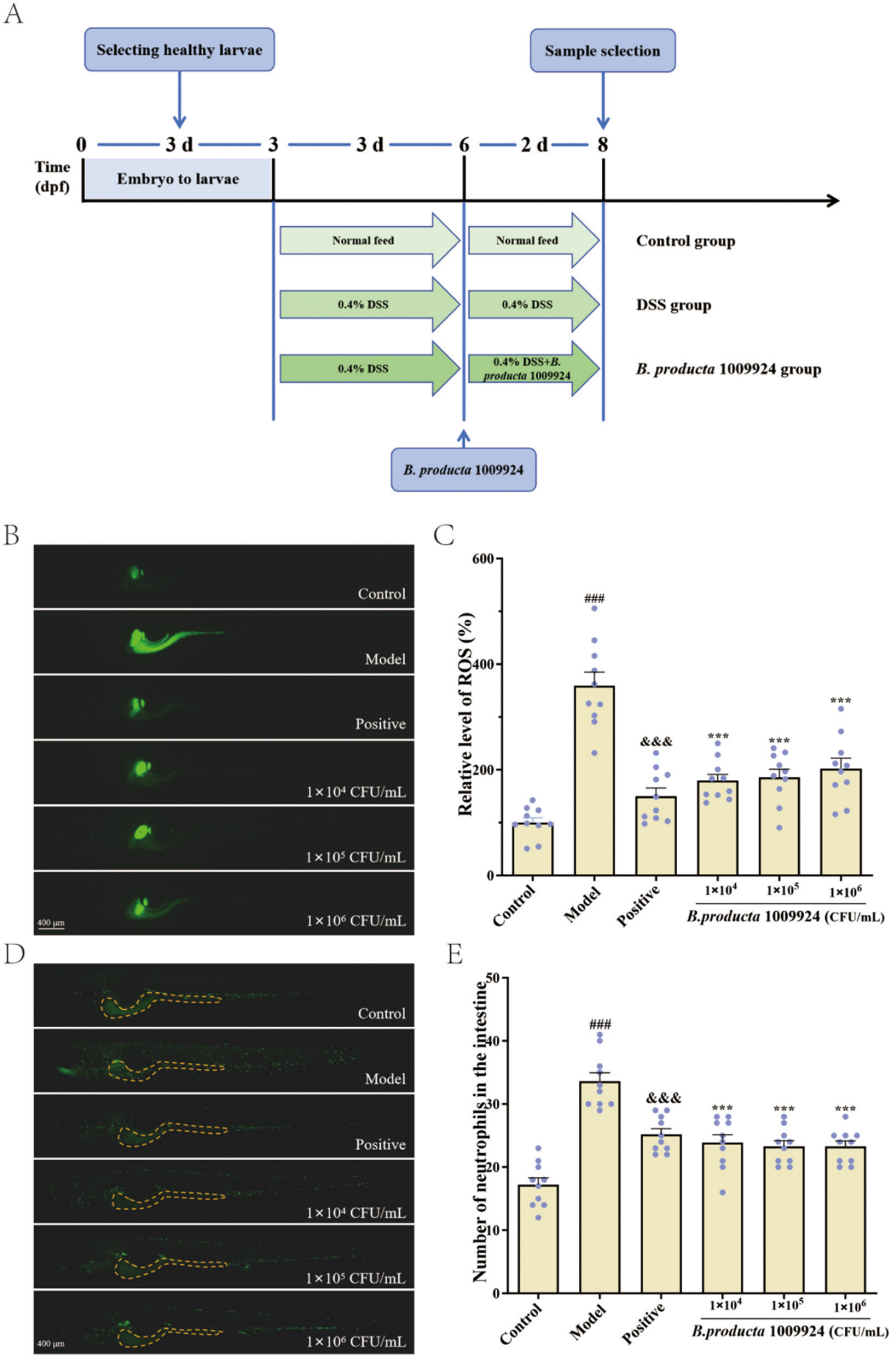
Alleviating effects of *B. producta* 1009924 on intestinal inflammation (*n* = 10). **(A)** Flowchart of experimental design for alleviating intestinal inflammation by *B. producta* 1009924. **(B)** Representative images of ROS levels in zebrafish. **(C)** Statistical analysis graph of ROS levels in zebrafish. **(D)** Representative images of neutrophils in zebrafish intestines. **(E)** Statistical results of the number of neutrophils in zebrafish intestines. All data were plotted as means ± SEM. Statistical significance was assessed using one-way ANOVA. Model vs. normal group: ^#^*p* < 0.05, ^##^*p* < 0.01, ^###^*p* < 0.001; positive vs. model group: ^&^*P* < 0.05, ^&&^*P* < 0.01, ^&&&^*P* < 0.001; 1009924 group vs. model: ^*^p < 0.05, **p < 0.01, ***p < 0.001. The same notation applies below.

Wild-type AB strain adult zebrafish and transgenic neutrophil green fluorescent adult zebrafish [Tg (mpx:EGFP) zebrafish] were maintained in a zebrafish breeding system (28.5°C, pH 7.5, conductivity 500–550 μS/cm, 14:10 h light–dark cycle). Zebrafish embryos were obtained through natural mating and incubated in E3 water (5 mM NaCl, 0.17 mM KCl, 0.33 mM CaCl_2_, and 0.33 mM MgSO_4_) and cultured in an incubator at a constant temperature of 28.5°C. The larvae were fed brine shrimp twice daily. At 3 days post-fertilization, wild-type AB strain zebrafish larvae and Tg (mpx:EGFP) larvae were randomly divided into 6 groups, with 510 zebrafish larvae per group, and incubated in 6-well plates. There were 6 treatments, namely, normal diet (control), 0.5% DSS (model group), 50 μg/mL mesalazine (postive group), 0.5% DSS + 1 × 10^4^ CFU/mL *B. producta* 1009924 (1 × 10^4^ CFU/mL group), 0.5% DSS + 1 × 10^5^ CFU/mL *B. producta* 1009924 (1 × 10^5^ CFU/mL group), and 0.5% DSS + 1 × 10^6^ CFU/mL *B. producta* 1009924 (1 × 10^6^ CFU/mL group). The control group was added with E3 culture water, while the other groups were treated with a 0.5% DSS solution, followed by incubation at 28.5°C for 72 h with fresh solutions replaced daily (Kim et al., 2012; Ni et al., 2023). Following model establishment, the aforementioned solutions were aspirated and discarded. The control group was then supplemented with 4 mL of E3 culture water, the model group with 4 mL of 0.5% DSS solution, and the positive group with 4 mL of a solution containing 0.5% DSS and 50 μg/mL mesalazine. For the 1009924 groups, the low-concentration group received 4 mL of bacterial suspension (1 × 104 CFU/mL) in 0.5% DSS, the medium-concentration group received 4 mL of bacterial suspension (1 × 105 CFU/mL) containing 0.5% DSS, and the high-concentration group received 4 mL of bacterial suspension (1 × 106 CFU/mL) in 0.5% DSS. All groups were incubated at 28.5°C for 48 h, with 5 mg of feed administered per well daily and the test solutions refreshed with freshly prepared aliquots daily, as described in Figure 1A. All the zebrafish experiments were approved by the Animal Welfare and Ethics Committee of Guangdong Human Microecology Engineering Technology Research Center's Laboratory (approval number: IACUC MC 0516-01-2024).

### 2.8 Neutrophil accumulation

Post-drug intervention, 10 larvae per group were randomly selected, rinsed thrice with E3 medium, and imaged under a fluorescence microscope to count intestinal neutrophils in Tg (mpx:EGFP) transgenic zebrafish, followed by statistical analysis (Zhao et al., 2023).

### 2.9 Reactive oxygen species (ROS) level detection

Following the intervention, 10 zebrafish larvae were randomly selected from each group and transferred to 6-well plates, with 5 mL of 5 μM DCFH-DA solution dispensed into each well. The larvae were incubated in the dark at 28.5°C for 1 h to allow probe loading, then rinsed three times with E3 embryo medium to remove unincorporated DCFH-DA, and observed under a fluorescence microscope (Leica DMi8). The statistical analysis of fluorescence intensity of individual zebrafish larvae was performed using the ImageJ software (Timothy and Kumar, 2024).

### 2.10 Histopathological analysis

#### 2.10.1 Hematoxylin and eosin staining

Three fish were selected from each group and immobilized with 4% paraformaldehyde at 4°C for 24 h, then processed according to standard procedures for hematoxylin-eosin (H&E) staining. The larvae were embedded in paraffin, cut into 3 μm sections, and stained with hematoxylin-eosin. Pathological changes in zebrafish liver tissue were observed under a microscope.

#### 2.10.2 Alcian blue staining

To assess intestinal goblet cell alterations, acid mucins were visualized via alcian blue staining. Zebrafish larvae were fixed in 4% paraformaldehyde at room temperature for 2 h, followed by dehydration and embedding. The sections were then stained with alcian blue following standardized procedures.

### 2.11 RNA isolation and reverse transcription-polymerase chain reaction (RT-PCR)

Post-intervention, 30 zebrafish larvae were randomly chosen from each group and euthanized to extract total RNA using Trizol reagent (Invitrogen, USA). First-strand cDNA synthesis was performed via reverse transcription with HiScript II Q RT SuperMix (Vazyme, China). Quantitative real-time PCR (qPCR) was then carried out on a StepOnePlus real-time fluorescent quantitative PCR system, using the synthesized cDNA as the template and ChamQ Universal SYBR qPCR Master Mix (Vazyme, China). The thermal cycling conditions were set as follows: initial denaturation at 95°C for 3 min; 40 cycles of denaturation at 95°C for 10 s, annealing at 60°C for 15 s, and extension at 60°C for 15 s; followed by a final extension step at 60°C for 2 min. The expression levels of target mRNAs were calculated using the 2^−ΔΔCt^ method by normalizing to glyceraldehyde-3-phosphate dehydrogenase (β-actin), and the corresponding primers are listed in Table 1.

**Table 1:** Real-time fluorescent quantitative PCR (qPCR) primers.

**Gene**	**Accession number**	**Sequences (5^′^-3^′^)**
*β-actin*	XM_030406939.1	F: GGTACCCATCTCCTGCTCCAA
		R: GAGCGTGGCTACTCCTTCACC
*IL-10*	NM_001020785.2	F: GCACTCCACAACCCCAATCG
		R:TGGCAAGAAAAGTACCTCTTGCAT
*IL-12*	AB183001	F: AGCAGGACTTGTTTGCTGGT
		R: TCCACTGCGCTGAAGTTAGA
*IL-6*	NM_001261449.1	F: TCAACTTCTCCAGCGTGATG
		R; CTTTCCCTCTTTTCCTCCTG
*TLR4*	NM_001131051	F: CGGCACTCCTCAAATCAACT
		R: GTCCTTCAAATCCTCCCACA
*MyD88*	NM_212814.2	ACCATCGCCAGTGAGCTTAT
		R: CAGATGGTCAGAAAGCGCAG
*NF-κB*	NM_001353873.1	F: AGTCAGCCTCAGATCCGTGTGTTT
		R: TTGTAAGCAAGGCCCATCAACTGC

### 2.12 Short-chain fatty acids (SCFAs) measurement

After the intervention, 30 zebrafish were randomly selected from each of the normal group, model group, and 1 × 104 CFU/mL *B. producta* 1009924 group for homogenization. The samples were centrifuged at 2,000 × *g* for 5 min at 4°C, and the supernatant and precipitate were collected separately. An 80% methanol solution was added to each sample tube, which were then vortexed and mixed, followed by high-speed centrifugation. The supernatant was pipetted and transferred to a 96-well plate (with the standard curve set up simultaneously). Two hundred milimeter 3-nitrophenylhydrazine (3-NPH) solution and 96 mM 1-(3-dimethylaminopropyl)-3-ethylcarbodiimide (EDC) solution were added in sequence. After sealing with a sealing film, the derivatization reaction was carried out at 40°C for 30 min, and then the plate was placed in a 20°C environment for cooling. Internal standard working solution and dichloromethane (DCM) were added, and oscillation extraction was performed for 10 min. After centrifugation, the organic phase was pipetted and transferred to a 96-well plate, which was then dried with nitrogen. The residue was redissolved in a 10% acetonitrile aqueous solution, vortexed, and then centrifuged; the supernatant was collected. The content of short-chain fatty acids in the supernatant was determined by liquid chromatography-tandem mass spectrometry (LC-MS/MS).

### 2.13 Statistical analysis

All the results were analyzed using GraphPad Prism 8.0. One-way ANOVA followed by Tukey's multiple comparisons test was used to compare differences among multiple groups. A *p*-value of < 0.05 was considered statistically significant. Data are presented as mean ± SEM.

## 3 Results

### 3.1 Fundamental characteristics of *B. producta* 1009924

**Figure 2:**
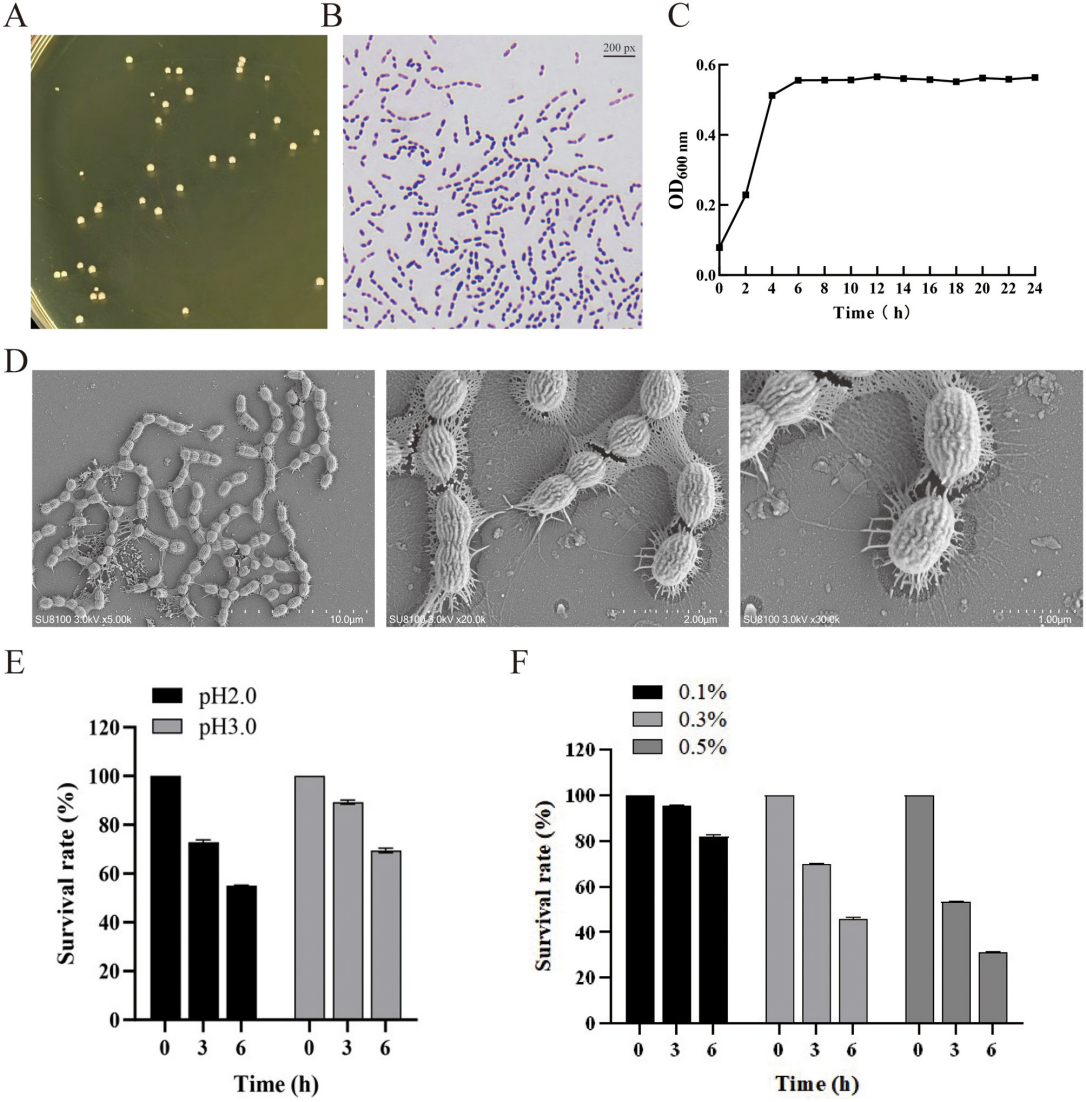
Morphological characteristics of *B. producta* 1009924. **(A)** Morphology of *B. producta* 1009924 on an agar plate; **(B)** Gram staining; **(C)** Growth curve graph (*n* = 3); **(D)** Submicroscopic structure; **(E)** Survival rate under different pH conditions; **(F)** Survival rate under different concentrations of bile salts.

*B. producta* 1009924 was isolated from a fresh fecal sample, and its 16S ribosomal RNA (rRNA) gene sequence is provided in Supplementary material. This strain is a strict anaerobe, forming circular, grayish-white colonies on BHI solid medium (Figure 2A). Gram staining revealed it is Gram-positive, with rod-shaped cells arranged in chains or pairs, featuring surface folds and pili (Figures 2B, D). Under anaerobic conditions at 37 °C, the logarithmic growth phase of *B. producta* 1009924 occurred between 2 and 6 h, followed by the stationary phase (Figure 2C). Gastric juice tolerance assays (Figure 2E) showed survival rates of (55.07 ± 0.23)% and (69.47 ± 0.93)% after 6 h of exposure to artificial gastric juice at pH 2.0 and pH 3.0, respectively. Bile salt tolerance tests indicated a concentration-dependent decline in survival: after 6 h incubation, survival rates were (81.8 ± 0.62)%, (45.81 ± 0.79)%, and (31.10 ± 0.21)% in the presence of 0.1, 0.3, and 0.5% (w/v) bile salts, respectively (Figure 2F).

### 3.2 Whole genome analysis of *B. producta* 1009924

**Figure 3:**
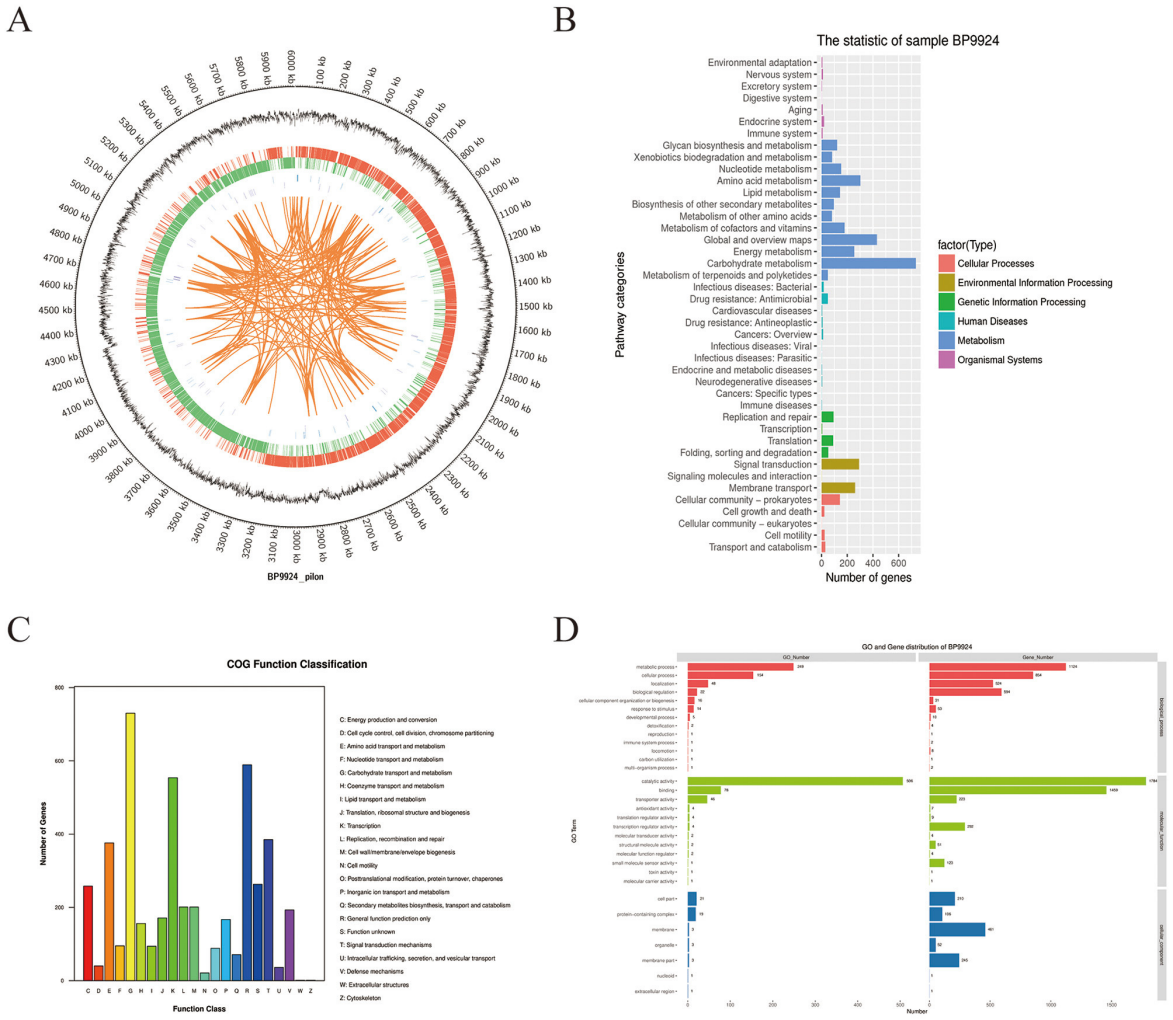
Whole-genome sequencing and bioinformatics analysis of *B. producta* 1009924. **(A)** Circular genome map: the outermost ring represents genomic location information, followed by (from outer to inner) GC content, coding genes on the positive strand (red), coding genes on the negative strand (green), non-coding information on the positive strand (blue), non-coding information on the negative strand (purple), and repeat sequence information in long segments of the genome (orange). **(B)** KEGG enrichment analysis. **(C)** COG enrichment analysis. **(D)** GO enrichment analysis.

As shown in Figure 3A and Table 2, the total genome length of *B. producta* 1009924 is 6,008,104 base pairs (bp), with a guanine (G) or cytosine (C) (i.e., GC) base content of 45.72%. It contains 5,214 coding genes, accounting for 89.16% of the total number of genes, and 122 non-coding RNAs, including 10 rRNAs and 64 tRNAs. Functional pathway annotation and statistical analysis of the genes of strain 1009924 using the KEGG database revealed that its genes are mainly concentrated in metabolism, environmental information processing, and genetic information processing. Among these, the metabolic pathways primarily include carbohydrate metabolism (734 genes), amino acid metabolism (302 genes), energy metabolism (255 genes), metabolism of cofactors and vitamins (180 genes), nucleotide metabolism (153 genes), lipid metabolism (144 genes), and biosynthesis and metabolism of glycans (122 genes) (Figure 3B). Consistent with the above findings, the COG database annotation results revealed that genes related to metabolic functions were primarily annotated to the transport and metabolism of carbohydrates, amino acids, nucleotides, and lipids (Figure 3C). The results of GO enrichment analysis revealed that the gene functions of *B. producta* 1009924 are significantly enriched in functional categories, including metabolic process, cellular process, biological regulation, cellular component organization or biogenesis, and response to stimulus, as well as catalytic activity, transport activity, and transcriptional regulation (Figure 3D). Furthermore, analysis results from the virulence factor database (VFDB) indicated that no virulence genes were identified in the genome of *B. producta* 1009924, confirming its biological safety. Notably, *B. producta* 1009924 harbors abundant gene clusters involved in lipid metabolism pathways and short-chain fatty acid metabolism pathways.

**Table 2:** Genomic information of *B. producta* 1009924.

**Feature**	**Value**
Size/bp	6,008,104
G+C content/%	45.72
Coding region/%	89.16
Total genes	5,336
RNA genes	74
rRNA genes	10
tRNA genes	64
Protein-coding genes	5,214
Protein-coding genes with enzymes	1,732
Genes with signal peptides	353
Genes with transmembrane helices	1,466

### 3.3 *B. producta* 1009924 reduces ROS levels induced by DSS

ROS levels in zebrafish were assessed via DCFH-DA fluorescent probe staining. As shown in Figures 1A, B, the relative ROS level in larvae of the normal group was (100.00 ± 9.33)%. After 72-h induction with 0.5% DSS, the fluorescent signal in zebrafish was significantly enhanced, with the relative ROS level rising significantly to (359.31 ± 25.69)% (*p* < 0.001), confirming the successful establishment of the intestinal inflammation model in zebrafish larvae. Compared to the model group, the positive control group and the *B. producta* 109924 groups at concentrations of 1 × 104, 1 × 105, and 1 × 106 CFU/mL all showed extremely significant reductions in relative ROS levels (*p* < 0.001), with values of (150.24 ± 15.33)%, (179.46 ± 11.87)%, (185.92 ± 15.15)%, and (202.37 ± 19.51)%, respectively.

### 3.4 *B. producta* 1009924 reduces neutrophil accumulation

As shown in Figures 1C, D, the transgenic neutrophil green fluorescent zebrafish Tg (mpx:EGFP) was used to observe the state of neutrophil aggregation in the intestine. The number of intestinal neutrophils in the normal group of juvenile zebrafish was (17.30 ± 1.05). After induction with 0.5% DSS, this number increased significantly to (33.30 ± 1.20) (*p* < 0.001), confirming the successful establishment of the intestinal inflammation model. Compared to the model group, the number of neutrophils in the positive group and each concentration group of *B. producta* 1009924 (1 × 104, 1 × 105, and 1 × 106 CFU/mL) decreased significantly (*p* < 0.001), with the numbers being (25.00 ± 0.82), (23.70 ± 1.19), (22.70 ± 0.73), and (23.40 ± 0.81), respectively.

### 3.5 *B. producta* 1009924 attenuated histological damage induced by DSS in zebrafish

**Figure 4:**
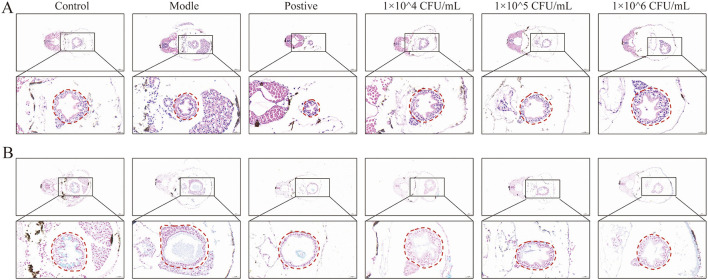
*B. producta* 1009924 protects the intestinal epithelium. **(A)** Hematoxylin and eosin (H&E) staining of zebrafish larval intestine. **(B)** Alcian Blue staining of zebrafish intestine, scale bars: 20 μm.

Hematoxylin-eosin (H&E) and Alcian blue staining were performed to assess intestinal histological characteristics in zebrafish larvae, encompassing intestinal villus architecture, intestinal epithelial integrity, and goblet cell morphological alterations. H&E staining results (Figure 4A) demonstrated that following 72-h exposure to 0.5% DSS, zebrafish intestines exhibited pathological manifestations including luminal dilation, intestinal wall thinning, villous atrophy (decreased quantity and shortened length), and nuclear dysplasia. Alcian blue staining (Figure 4B) revealed a significant reduction in intestinal acidic mucin secretion in the model group. Compared to the model group, the extent of pathological damage was decreased significantly in the three *B. producta* 1009924 and mesalazine (positive) group. Notably, the 1 × 106 CFU/mL *B. producta* 1009924 treatment group displayed intestinal architecture analogous to the normal control group. In contrast, the secretion level of acidic mucin failed to revert to the control level.

### 3.6 *B. producta* 1009924 decreased inflammatory cytokine expression in zebrafish with DSS-induced colitis

**Figure 5:**
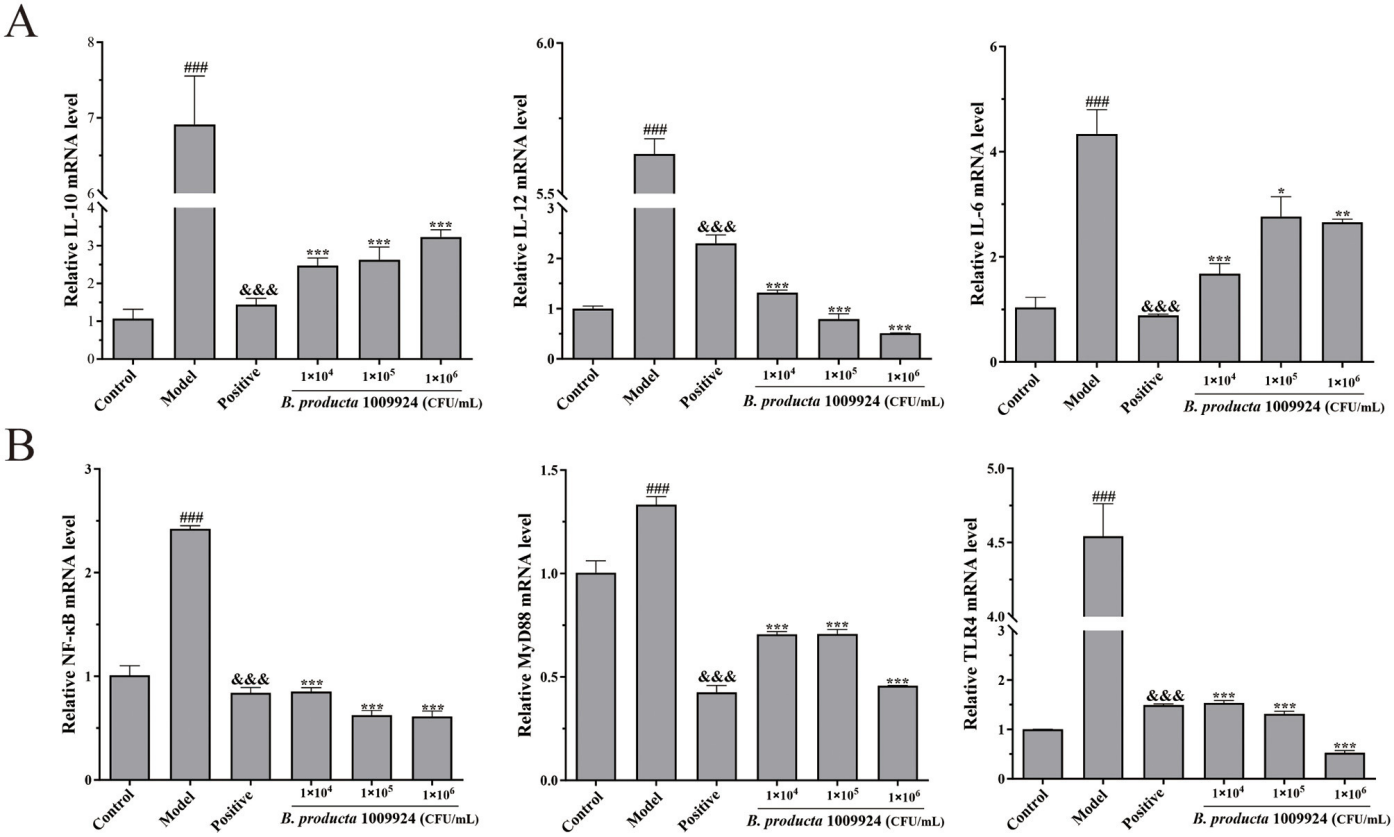
**(A)** *B. producta* 1009924 inhibits inflammatory cytokine production in DSS-induced colitis (*n* = 3). The mRNA expression levels of *IL-10, IL-12*, and *IL-6*. **(B)** Gene expression levels of inflammation-related pathway genes (*TLR4, MyD88*, and *NF-*κ*B*).

As shown in Figure 5A, the expression levels of immunomodulatory cytokines (*IL-10* and *IL-12*) and the proinflammatory cytokine (*IL-6*) were significantly upregulated (*p* < 0.01) in zebrafish subjected to DSS-induced intestinal inflammation (model group). In contrast, *B. producta* 1009924 treatment (1 × 104, 1 × 105, and 1 × 106 CFU/mL) dose-dependently attenuated the expression of these cytokines post-DSS induction (*p* < 0.05). *TLR4, MyD88*, and *NF-*κ*B* are key genes in the TLR4/NF-κB pathway associated with colitis. As shown in Figure 5B, the expression levels of these three genes were significantly elevated in the model group (*P* < 0.01). Conversely, both the positive control and *B. producta* 1009924-treated groups (1 × 104−1 × 106 CFU/mL) demonstrated profound suppression of TLR4/MyD88/NF-κB axis activation (*p* < 0.001).

### 3.7 The impact of *B. producta* 1009924 on SCFA

**Figure 6:**
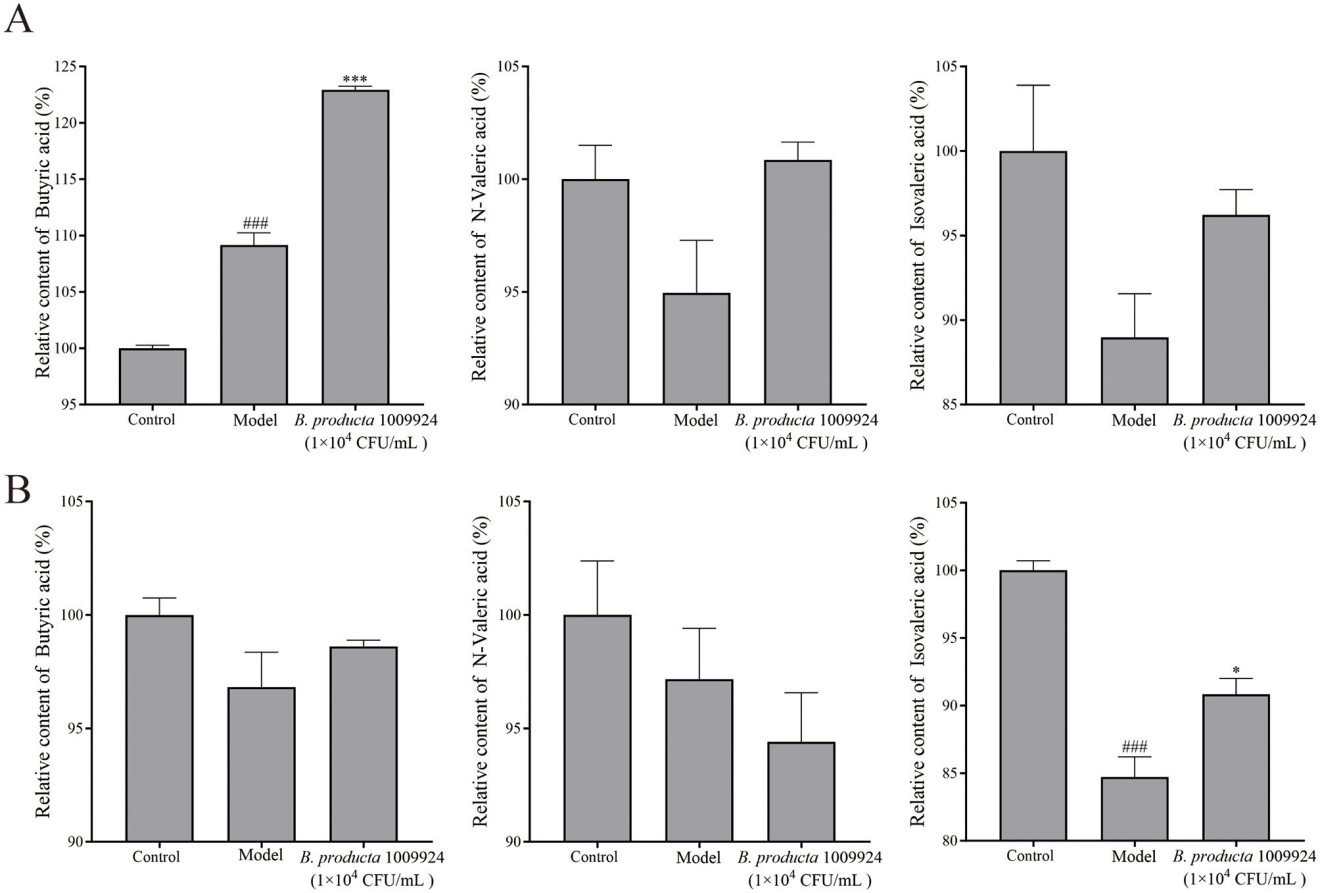
Effect of *B. producta* 1009924 on short-chain fatty acid content in zebrafish (*n* = 3). **(A)** The contents of SCFAs (butyric acid, valeric acid, isopentyl acid) of the supernatant. **(B)** The contents of SCFAs in zebrafish sediment.

At the end of the experiment, zebrafish in the normal group, model group, and 1 × 104 CFU/mL *B. producta* 1009924 group were homogenized, with supernatants and precipitates separated to detect short-chain fatty acids (butyric acid, valeric acid, and isovaleric acid). In supernatants (Figure 6A): compared to the normal group, the model group showed significantly increased butyric acid (*p* < 0.01) and decreased valeric acid and isovaleric acid (*p* > 0.05); compared to the model group, the 1009924 group had significantly increased butyric acid (*p* < 0.001), and elevated valeric acid and isovaleric acid without statistical significance (*p* > 0.05). In precipitates (Figure 6B): compared to the normal group, the model group had decreased butyric acid and valeric acid (*p* > 0.05) and significantly decreased isovaleric acid (*p* < 0.001); compared to the model group, the 1009924 group showed slightly increased butyric acid and slightly decreased valeric acid (both *p* > 0.05), while isovaleric acid was significantly increased (*p* < 0.05).

## 4 Discussion

*B. producta* is an anaerobic microorganism with probiotic properties that is widely present in the human intestine and accounts for a relatively high abundance in the gastrointestinal microbiota (Zhang et al., 2019; Xing et al., 2024). In this study, the strain *Blautia producta* 1009924 was isolated from fresh fecal samples, and its biological characteristics are consistent with those of other *B. producta* strains (Liu et al., 2021): it is Gram-positive, with bacterial cells arranged in pairs or chains. Growth curve analysis showed that the strain enters the logarithmic growth phase between 2 and 6 h, followed by the stationary phase. Genomic analysis revealed that the genome of *B. producta* 1009924 is 6.05 Mb in length and contains 5,214 coding genes. Functional enrichment indicated that these genes are mainly involved in the transport and metabolism of amino acids, nucleotides, carbohydrates, coenzymes, lipids, inorganic ions, and secondary metabolites. Additionally, they participate in biological processes such as catalytic activity, cell cycle regulation, translation, transcription, cell wall/membrane/envelope biogenesis, signal transduction, intracellular transport, secretion, vesicle trafficking, and defense mechanisms.

In patients with intestinal diseases, the abundance of *B. producta* is significantly reduced, showing a negative correlation with inflammatory bowel disease (Chen et al., 2014, 2012). During intestinal inflammation, neutrophils are recruited to the site of inflammation; functionally abnormal neutrophils infiltrate and activate in the intestine, further exacerbating tissue damage by releasing inflammatory factors (Shang et al., 2012; de Bruyn et al., 2014). Additionally, patients with intestinal inflammation often exhibit imbalanced oxidative stress, characterized by excessive elevation of ROS levels. This not only interferes with immune responses, damages cellular components, and induces tissue injury (Lushchak, 2014) but also, through interactions with tumor-related genes, excessive ROS can ultimately contribute to the development of intestinal cancer (Ran et al., 2017).

In this study, the intestinal inflammation model induced by DSS exhibited typical pathological features: substantial accumulation of ROS, abnormal aggregation of neutrophils in the intestine, obvious intestinal tissue damage, reduced secretion of acidic mucin, significantly increased expression levels of the pro-inflammatory factor IL-6 and immune factors IL-10 and IL-12, and activation of the TLR4/NF-κB signaling pathway, ultimately leading to the development of intestinal inflammation in zebrafish. The intervention results showed that treatment with *B. producta* 1009924 fermentation broth at concentrations of 1 × 104 CFU/mL, 1 × 105 CFU/mL, and 1 × 106 CFU/mL could reduce intestinal ROS levels in zebrafish with intestinal inflammation, alleviate neutrophil infiltration, regulate inflammatory and immune responses, protect intestinal tissue integrity, and thereby exert a role in alleviating intestinal inflammation. Further observations revealed that this strain exerted a certain reparative effect on inflamed intestinal tissues, as evidenced by an increase in the height and quantity of intestinal villi; however, no effect on restoring the secretion of intestinal acidic mucin was observed. At the mechanistic level, the regulatory effect of *B. producta* 1009924 on intestinal inflammation may be associated with the downregulation of *IL-6, IL-10*, and *IL-12* expression. Its anti-inflammatory effect is achieved by inhibiting the activation of the colitis-related TLR4/NF-κB signaling pathway.

Previous studies have demonstrated that *B. producta* can maintain intestinal microenvironment homeostasis and prevent inflammation through pathways such as upregulating intestinal regulatory T cells and producing SCFAs (Kim et al., 2014). As key metabolites from microbial fermentation of carbohydrates in the colon, SCFAs (including butyric acid, valeric acid, isovaleric acid) not only serve as the main energy source for intestinal epithelial cells but also play an irreplaceable role in maintaining the structural stability of the intestinal microbiota and enhancing the intestinal mucosal barrier function (Tramontano et al., 2018). As intestinal microbiota metabolites, SCFAs can both activate cellular receptors (Singh et al., 2014) and act as HDAC inhibitors (Chang et al., 2014; Rooks and Garrett, 2016) to inhibit the TLR4/NF-κB pathway, downregulate inflammatory factors such as IL-6 (Zhang et al., 2011; Rooks and Garrett, 2016), thereby exerting anti-inflammatory effects. In this study, the DSS-induced intestinal inflammation model exhibited typical SCFA metabolic disorders: the content of butyric acid was abnormally elevated (which may be related to abnormal fermentation caused by intestinal microbiota imbalance), the content of isovaleric acid was significantly reduced, and valeric acid showed a decreasing trend. This suggests that the imbalance of the SCFA profile may be involved in the pathological process of inflammation. In sharp contrast, after intervention with *B. producta* 1009924 fermentation broth, the contents of butyric acid, valeric acid, and isovaleric acid in the intestines of model zebrafish all showed a trend of normalization—not only reversed the decrease in isovaleric acid and valeric acid, but also potentially restored the physiological function of butyric acid by optimizing its abnormally high expression. This result echoes the previously observed effect that “the strain inhibits the activation of the TLR4/NF-κB pathway and downregulates inflammatory factors such as IL-6,” suggesting that *B. producta* 1009924 may regulate intestinal inflammation through the “SCFAs-HDAC-TLR4/NF-κB” axis by reshaping the SCFAs metabolic profile, thus providing a metabolic-level mechanistic explanation for its role in alleviating intestinal damage.

## Data Availability

The datasets presented in this study can be found in online repositories. The names of the repository/repositories and accession number(s) can be found below: https://www.ncbi.nlm.nih.gov/, PRJNA1209333.

## References

[B1] Chang P. V. Hao L. Offermanns S. Medzhitov R. (2014). The microbial metabolite butyrate regulates intestinal macrophage function via histone deacetylase inhibition. Proc. Natl. Acad. Sci. U. S. A. 111, 2247–2252. 10.1073/pnas.1322269111 24390544 PMC3926023

[B2] Chen L. Wang W. Zhou R. Ng S. C. Li J. Huang M.. (2014). Characteristics of fecal and mucosa-associated microbiota in Chinese patients with inflammatory bowel disease. Medicine 93:e51. 10.1097/MD.0000000000000051 25121355 PMC4602441

[B3] Chen M. Liu C. Dai M. Wang Q. Li C. Hung W.. (2022). Bifidobacterium lactis BL-99 modulates intestinal inflammation and functions in zebrafish models. PLoS ONE 17:e0262942. 10.1371/journal.pone.0262942 35171916 PMC9126502

[B4] Chen W. Liu F. Ling Z. Tong X. Xiang C. (2012). Human intestinal lumen and mucosa-associated microbiota in patients with colorectal cancer. PLoS ONE 7:e39743. 10.1371/journal.pone.0039743 22761885 PMC3386193

[B5] de Bruyn M. Arijs I. Wollants W. J. Machiels K. Van Steen K. Van Assche G.. (2014). Neutrophil gelatinase B-associated lipocalin and matrix metalloproteinase-9 complex as a surrogate serum marker of mucosal healing in ulcerative colitis. Inflamm. Bowel Dis. 20, 1198–1207. 10.1097/MIB.0000000000000068 24871805

[B6] Delcher A. L. Bratke K. A. Powers E. C. Salzberg S. L. (2007). Identifying bacterial genes and endosymbiont DNA with Glimmer. Bioinformatics 23, 673–679. 10.1093/bioinformatics/btm009 17237039 PMC2387122

[B7] Harris M. A. Clark J. Ireland A. Lomax J. Ashburner M. Foulger R.. (2004). The Gene Ontology (GO) database and informatics resource. Nucleic Acids Res. 32, D258–D261. 10.1093/nar/gkh036 14681407 PMC308770

[B8] Huang J. Zhang W. Hu Z. Liu Z. Du T. Dai Y.. (2020). Isolation, characterization and selection of potential probiotic lactic acid bacteria from feces of wild boar, native pig and commercial pig. Livestock Sci. 237:104036. 10.1016/j.livsci.2020.104036

[B9] Kim C. H. Park J. Kim M. (2014). Gut microbiota-derived short-chain fatty acids, T cells, and inflammation. Immune Netw. 14, 277–288. 10.4110/in.2014.14.6.277 25550694 PMC4275385

[B10] Kim J. J. Shajib M. S. Manocha M. M. Khan W. I. (2012). Investigating intestinal inflammation in DSS-induced model of IBD. J. Vis. Exp. 3678. 10.3791/3678 22331082 PMC3369627

[B11] Kokkinosa A. Fasseas C. Eliopoulos E. Kalantzopoulos G. J. L. L. (1998). Cell size of various lactic acid bacteria as determined by scanning electron microscope and image Analysis 78, 491–500. 10.1051/lait:1998546

[B12] Liu C. Finegold S. M. Song Y. Lawson P. A. (2008). Reclassification of *Clostridium coccoides, Ruminococcus hansenii, Ruminococcus hydrogenotrophicus, Ruminococcus luti, Ruminococcus productus* and *Ruminococcus schinkii* as *Blautia coccoides* gen. nov., comb. nov., *Blautia hansenii* comb. nov., *Blautia hydrogenotrophica* comb. nov., *Blautia luti* comb. nov., *Blautia producta* comb. nov., *Blautia schinkii* comb. nov. and description of *Blautia wexlerae* sp. nov., isolated from human faeces. Int. J. Syst. Evol. Microbiol. 58(Pt. 8), 1896–1902. 10.1099/ijs.0.65208-0 18676476

[B13] Liu W. Zhang Y. Qiu B. Fan S. Ding H. Liu Z.. (2018). Quinoa whole grain diet compromises the changes of gut microbiota and colonic colitis induced by dextran Sulfate sodium in C57BL/6 mice. Sci. Rep. 8:14916. 10.1038/s41598-018-33092-9 30297695 PMC6175902

[B14] Liu X. Mao B. Gu J. Wu J. Cui S. Wang G.. (2021). Blautia-a new functional genus with potential probiotic properties? Gut Microbes 13, 1–21. 10.1080/19490976.2021.1875796 33525961 PMC7872077

[B15] Lorowitz W. H. Bryant M. P. (1984). *Peptostreptococcus* productus strain that grows rapidly with CO as the energy source. Appl. Environ. Microbiol. 47, 961–964. 10.1128/aem.47.5.961-964.1984 6430231 PMC240027

[B16] Lushchak V. I. (2014). Free radicals, reactive oxygen species, oxidative stress and its classification. Chem. Biol. Interact. 224, 164–175. 10.1016/j.cbi.2014.10.016 25452175

[B17] Mao B. Guo W. Cui S. Zhang Q. Zhao J. Tang X.. (2024). *Blautia producta* displays potential probiotic properties against dextran sulfate sodium-induced colitis in mice. Food Sci. Hum. Wellness 13, 709–720. 10.26599/FSHW.2022.9250060

[B18] McCarthy A. (2010). Third generation DNA sequencing: pacific biosciences' single molecule real time technology. Chem. Biol. 17, 675–676. 10.1016/j.chembiol.2010.07.004 20659677

[B19] Molodecky N. A. Soon I. S. Rabi D. M. Ghali W. A. Ferris M. Chernoff G.. (2012). Increasing incidence and prevalence of the inflammatory bowel diseases with time, based on systematic review. Gastroenterology 142, 46–54.e42. Quiz e30. 10.1053/j.gastro.2011.10.001 22001864

[B20] Ni Y. Zhang Y. Zheng L. Rong N. Yang Y. Gong P.. (2023). *Bifidobacterium* and *Lactobacillus* improve inflammatory bowel disease in zebrafish of different ages by regulating the intestinal mucosal barrier and microbiota. Life Sci. 324:121699. 10.1016/j.lfs.2023.121699 37061125

[B21] Nurk S. Walenz B. P. Rhie A. Vollger M. R. Logsdon G. A. Grothe R.. (2020). HiCanu: accurate assembly of segmental duplications, satellites, and allelic variants from high-fidelity long reads. Genome Res. 30, 1291–1305. 10.1101/gr.263566.120 32801147 PMC7545148

[B22] Ogata H. Goto S. Sato K. Fujibuchi W. Bono H. Kanehisa M.. (1999). KEGG: kyoto encyclopedia of genes and genomes. Nucleic Acids Res. 27, 29–34. 10.1093/nar/27.1.29 9847135 PMC148090

[B23] Ran T. Geng S. Li L. (2017). Neutrophil programming dynamics and its disease relevance. Sci. China Life Sci. 60, 1168–1177. 10.1007/s11427-017-9145-x 28971361

[B24] Rooks M. G. Garrett W. S. (2016). Gut microbiota, metabolites and host immunity. Nat. Rev. Immunol. 16, 341–352. 10.1038/nri.2016.42 27231050 PMC5541232

[B25] Shang K. Bai Y. P. Wang C. Wang Z. Gu H. Y. Du X.. (2012). Crucial involvement of tumor-associated neutrophils in the regulation of chronic colitis-associated carcinogenesis in mice. PLoS ONE 7:e51848. 10.1371/journal.pone.0051848 23272179 PMC3525572

[B26] Singh N. Gurav A. Sivaprakasam S. Brady E. Padia R. Shi H.. (2014). Activation of Gpr109a, receptor for niacin and the commensal metabolite butyrate, suppresses colonic inflammation and carcinogenesis. Immunity 40, 128–139. 10.1016/j.immuni.2013.12.007 24412617 PMC4305274

[B27] Timothy R. Kumar I. P. (2024). Comparative estimation of ROS levels using DCFDA in zebrafish larvae model on the treatment of camphene and doxorubicin for antioxidant property. E3S Web Conf. 477:00050. 10.1051/e3sconf/202447700050

[B28] Tramontano M. Andrejev S. Pruteanu M. Klünemann M. Kuhn M. Galardini M.. (2018). Nutritional preferences of human gut bacteria reveal their metabolic idiosyncrasies. Nat. Microbiol. 3, 514–522. 10.1038/s41564-018-0123-9 29556107

[B29] Wang Z. K. Yang Y. S. (2013). Upper gastrointestinal microbiota and digestive diseases. World J. Gastroenterol. 19, 1541–1550. 10.3748/wjg.v19.i10.1541 23539678 PMC3602471

[B30] Xing H. Zhang Y. Krämer M. Kissmann A.-K. Henkel M. Weil T.. (2022). A polyclonal selex aptamer library directly allows specific labelling of the human gut bacterium *Blautia producta* without isolating individual aptamers. Molecules 27:5693. 10.3390/molecules27175693 36080459 PMC9458011

[B31] Xing H. Zhang Y. Li R. Ruzicka H. M. Hain C. Andersson J.. (2024). A *Blautia producta* specific gFET-based aptasensor for quantitative monitoring of microbiome quality. Nanoscale Horiz. 10:124134. 10.1039/d4nh00281d 39420595

[B32] Yang J. Bindels L. B. Segura Munoz R. R. Martínez I. Walter J. Ramer-Tait A. E.. (2016). Disparate metabolic responses in mice fed a high-fat diet supplemented with maize-derived non-digestible feruloylated oligo- and polysaccharides are linked to changes in the gut microbiota. PLoS ONE 11:e0146144. 10.1371/journal.pone.0146144 26731528 PMC4701460

[B33] Zhang W. Li J. Lu S. Han N. Miao J. Zhang T.. (2019). Gut microbiota community characteristics and disease-related microorganism pattern in a population of healthy Chinese people. Sci. Rep. 9:1594. 10.1038/s41598-018-36318-y 30733472 PMC6367356

[B34] Zhang W. H. Jiang Y. Zhu Q. F. Gao F. Dai S. F. Chen J.. (2011). Sodium butyrate maintains growth performance by regulating the immune response in broiler chickens. Br. Poult. Sci. 52, 292–301. 10.1080/00071668.2011.578121 21732874

[B35] Zhao Q. Chang H. Zheng J. Li P. Ye L. Pan R.. (2023). A novel Trmt5-deficient zebrafish model with spontaneous inflammatory bowel disease-like phenotype. Signal Transduct Target Ther. 8:86. 10.1038/s41392-023-01318-6 36849517 PMC9971238

[B36] Zoetendal E. G. Collier C. T. Koike S. Mackie R. I. Gaskins H. R. (2004). Molecular ecological analysis of the gastrointestinal microbiota: a review. J. Nutr. 134, 465–472. 10.1093/jn/134.2.465 14747690

