# Supplementary material for: The mitigative effects of Blautia producta 1009924 on DSS-induced intestinal inflammation
Source: Front Microbiol. 2025 Aug 8;16:1560441. doi: 10.3389/fmicb.2025.1560441 (PMC12370734; doi:10.3389/fmicb.2025.1560441)
Supplement: Supplementary file 1 [file Data_Sheet_1.docx]

***Effects of blank medium on Intestinal Inflammation***

Because *B. producta* 1009924 fermentation broth contains medium, we set up a blank medium control group and detected the number of neutrophils and ROS levels. The number of neutrophils in the intestinal tract of zebrafish in the blank medium group was 29.20 ± 0.97, which was lower than that in the model group and had statistical difference (*P <* 0.05) (Fig.S1a). But the number of neutrophils in the blank medium group was higher than that in the *B. producta* 1009924 fermentation broth group. DCFH-DA staining was employed to detect the ROS levels in zebrafish. The relative ROS level of zebrafish in the blank medium group was 279.45 ± 37.95 % , which was lower than that in the model group, but the difference was not statistically significant (*P* > 0.05) (Fig.S1b).The above results indicate that because the medium contains raw materials with anti-inflammatory effects, it can alleviate intestinal inflammation to a certain extent, but the effect of the *B. producta* 1009924 fermentation broth group on alleviating intestinal inflammation is more significant.


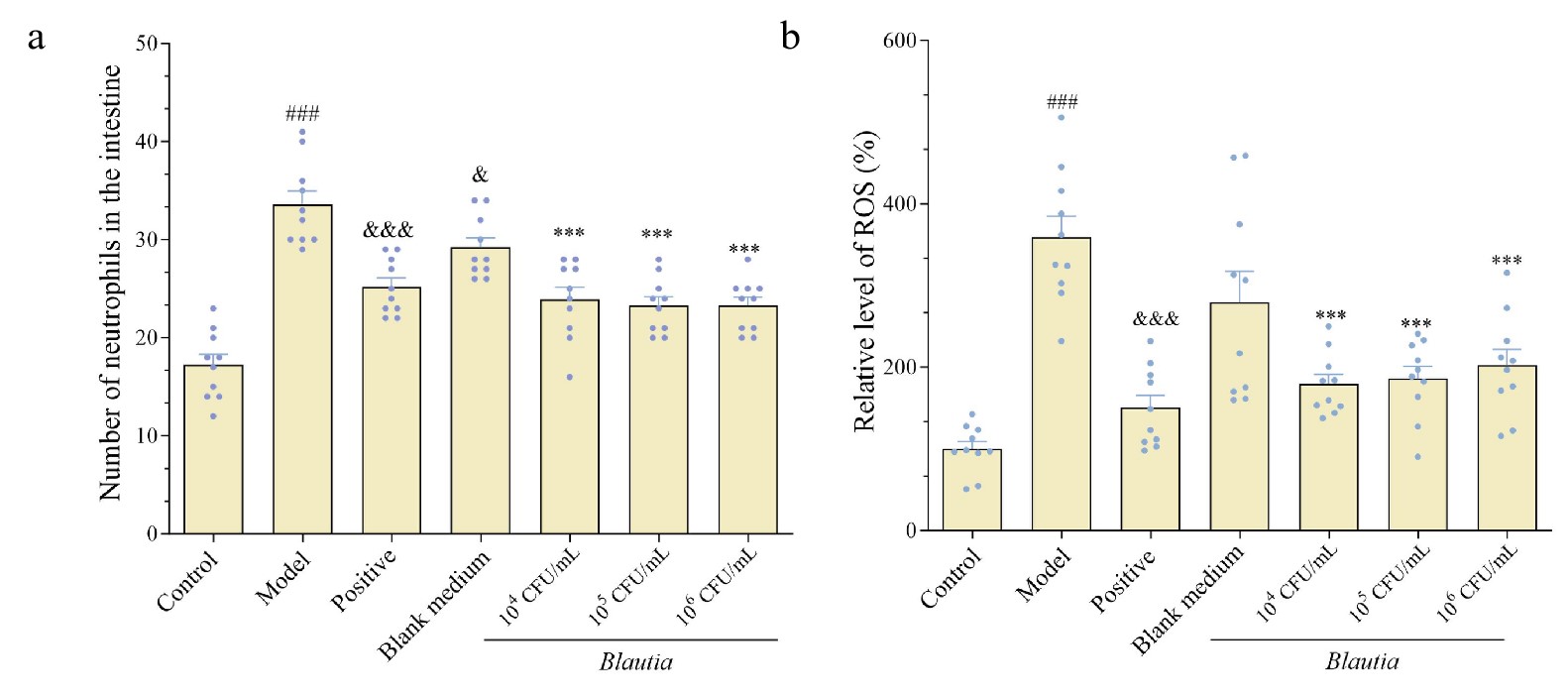


Fig.S1 Alleviating effects of blank medium on intestinal inflammation: (a)Statistical results of ROS levels in zebrafish in each group; (b) Statistical results of neutrophil counts in zebrafish intestines in each group. Experimental data are presented as mean ± SEM and analyzed. Based on t-test, as compared to the control group, ^#^*P* < 0.05，^##^*P* < 0.01，^###^*P* < 0.001; as compared to the model group, ^&^*P* < 0.05，^&&^*P* < 0.01，^&&&^*P* < 0.00. Based on the One-way ANOVA analysis, as compared to the model group, ^*^*P* < 0.05，^**^*P* < 0.01，^***^*P* < 0.001.
